# Genetics and Crime: Integrating New Genomic Discoveries Into Psychological Research About Antisocial Behavior

**DOI:** 10.1177/0956797617744542

**Published:** 2018-03-07

**Authors:** J. Wertz, A. Caspi, D. W. Belsky, A. L. Beckley, L. Arseneault, J. C. Barnes, D. L. Corcoran, S. Hogan, R. M. Houts, N. Morgan, C. L. Odgers, J. A. Prinz, K. Sugden, B. S. Williams, R. Poulton, T. E. Moffitt

**Affiliations:** 1 Department of Psychology & Neuroscience, Duke University; 2 Department of Psychiatry & Behavioral Sciences, Duke University School of Medicine; 3 Center for Genomic and Computational Biology, Duke University; 4 Social, Genetic, & Developmental Psychiatry Research Centre, Institute of Psychiatry, Psychology, & Neuroscience, King’s College London; 5 Department of Medicine, Duke University School of Medicine; 6 Social Science Research Institute, Duke University; 7 Demography Unit, Department of Sociology, Stockholm University; 8 School of Criminal Justice, University of Cincinnati; 9 Dunedin Multidisciplinary Health and Development Research Unit, Department of Psychology, University of Otago; 10 Home Office, London, United Kingdom; 11 Sanford School of Public Policy, Duke University

**Keywords:** crime, genetics, antisocial behavior, longitudinal

## Abstract

Drawing on psychological and sociological theories of crime causation, we tested the hypothesis that genetic risk for low educational attainment (assessed via a genome-wide polygenic score) is associated with criminal offending. We further tested hypotheses of how polygenic risk relates to the development of antisocial behavior from childhood through adulthood. Across the Dunedin and Environmental Risk (E-Risk) birth cohorts of individuals growing up 20 years and 20,000 kilometers apart, education polygenic scores predicted risk of a criminal record with modest effects. Polygenic risk manifested during primary schooling in lower cognitive abilities, lower self-control, academic difficulties, and truancy, and it was associated with a life-course-persistent pattern of antisocial behavior that onsets in childhood and persists into adulthood. Crime is central in the nature-nurture debate, and findings reported here demonstrate how molecular-genetic discoveries can be incorporated into established theories of antisocial behavior. They also suggest that improving school experiences might prevent genetic influences on crime from unfolding.

Advances in the genomic sciences are now making it possible to investigate genetic influences on behavior at the molecular-genetic level, using genome-wide association studies (Visscher et al., 2017). Although effect sizes for individual genetic variants revealed in such studies are tiny, it is possible to aggregate the effects of millions of variants across the genome to construct polygenic scores, which index a person’s position on a continuum of genetic propensity toward specific phenotypes (Dudbridge, 2013). Associations between individuals’ polygenic scores and behaviors are nondeterministic, with a polygenic score on the higher or lower end of the continuum slightly increasing or decreasing the odds of an outcome. One of the largest and most successful genome-wide association studies for a social-science outcome has been conducted for educational attainment (Okbay et al., 2016). Because educational attainment is a central phenotype in the nomological net of constructs in the psychological and social sciences, genetic discoveries for education may have implications for research and theory about outcomes that are known to be linked to education. Here, we tested the hypothesis that polygenic influences on educational attainment predict criminal offending.

We derived the hypothesis that polygenic influences on educational attainment predict criminal offending by integrating genetic discoveries about educational attainment with established theories about crime. First, individuals with lower polygenic scores for educational attainment tend to complete less schooling, which is a correlate of criminal offending (Stattin & Magnusson, 1995). Truncated education may leave people with fewer legitimate methods to achieve wealth or status, increasing incentives to pursue crime (Agnew, 1992; Merton, 1938). Second, polygenic scores for educational attainment partly reflect early-emerging traits that affect success in school, such as cognitive ability and self-control (Belsky et al., 2016); these traits also increase risk for crime (Gottfredson & Hirschi, 1990; Moffitt, 1993b). Third, low polygenic scores for education may predict poor school performance and academic frustration, which reduce a protective factor that helps deter young people from crime, namely, commitment to school and its social norms (Catalano, Oesterle, Fleming, & Hawkins, 2004).

On the basis of these considerations, we tested the hypothesis that molecular-genetic predictors of educational attainment would forecast individuals’ criminal offending. We tested this hypothesis in two birth cohorts comprising nearly 3,000 participants. In both cohorts, we linked genetic data to official police records. We additionally investigated whether the effects of polygenic scores on criminal offending would survive after accounting for two significant markers of a criminogenic family environment: growing up in socioeconomic deprivation and having antisocial parents.

Our developmental study examined how the association between genetic influences on educational attainment and criminal offending emerges over time and in concert with (and independently of) educational attainment itself. First, we examined early-emerging psychological and behavioral risk factors that may connect genetic differences between individuals to their risk of criminal offending. Second, we tested the hypothesis that genetic influences would be particularly strong among individuals who show a pattern of antisocial behavior that begins in childhood and thereafter follows a persistent pattern into adulthood, often referred to as “life-course persistent” antisocial behavior (Moffitt, 1993a).

## Method

### Samples

#### Environmental Risk (E-Risk) cohort

**Fig. 1.**
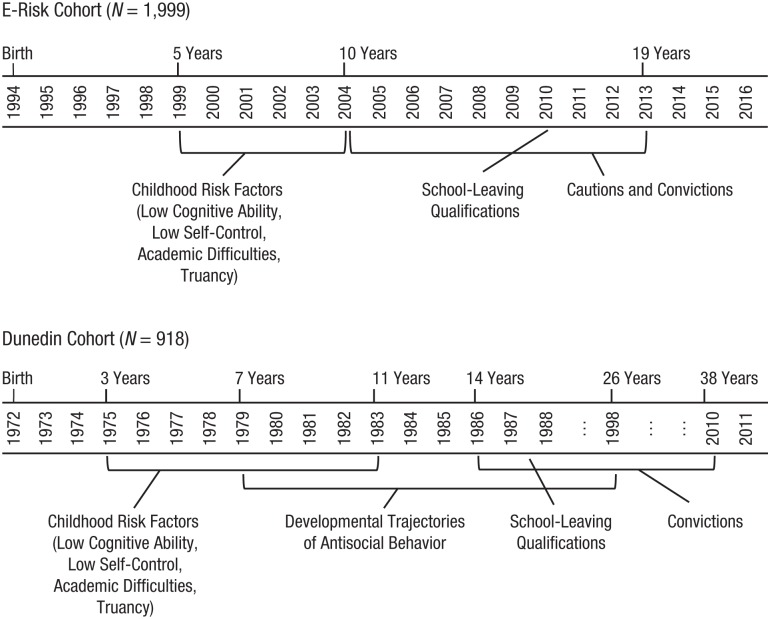
Study timelines of the Environmental Risk (E-Risk) and Dunedin cohorts. The figure depicts the observation period of early-emerging psychological and behavioral risk factors (low cognitive ability, low self-control, academic difficulties in primary school, and truancy), school-leaving qualifications, and crime records (cautions and convictions) in the two cohorts and developmental trajectories of antisocial behavior in the Dunedin cohort.

Participants in the first cohort were members of the E-Risk Longitudinal Twin Study, which tracks the development of a birth cohort of 2,232 British participants (Fig. 1). The sample was drawn from a larger birth register of twins born in England and Wales in 1994 and 1995 (Trouton, Spinath, & Plomin, 2002). Full details about the sample are reported elsewhere (Moffitt & E-Risk Study Team, 2002). Briefly, the E-Risk sample was constructed from 1999 to 2000, when 1,116 families (93% of those eligible) with same-sex 5-year-old twins participated in home-visit assessments. This sample consisted of 56% monozygotic (MZ) and 44% dizygotic (DZ) twin pairs; sex was evenly distributed within zygosity (49% male). Families were recruited to represent the UK population of families with newborns in the 1990s on the basis of residential location throughout England and Wales and mother’s age. Teenage mothers with twins were overselected to replace high-risk families who were selectively lost to the register through nonresponse. Older mothers who had twins via assisted reproduction were underselected to avoid an excess of well-educated older mothers. The study sample represents the full range of socioeconomic conditions in the United Kingdom, as reflected in the families’ distribution on a neighborhood-level socioeconomic index (A Classification of Residential Neighbourhoods, or ACORN, developed by CACI for commercial use; Odgers, Caspi, Bates, Sampson, & Moffitt, 2012): 25.6% of E-Risk families live in “wealthy-achiever” neighborhoods, compared with 25.3% nationwide; 5.3% versus 11.6% live in “urban-prosperity” neighborhoods; 29.6% versus 26.9% live in “comfortably-off” neighborhoods; 13.4% versus 13.9% live in “moderate-means” neighborhoods; and 26.1% versus 20.7% live in “hard-pressed” neighborhoods. E-Risk underrepresents “urban-prosperity” neighborhoods because such households are likely to be childless.

Home-visit assessments took place when participants were aged 5, 7, 10, 12, and, most recently, 18 years, when 93% of the participants took part. At ages 5, 7, 10, and 12 years, assessments were carried out with participants as well as their mothers (or primary caretakers); the home visit at age 18 included interviews only with participants. Each twin was assessed by a different interviewer. These data are supplemented by searches of official records and by questionnaires that are mailed, as developmentally appropriate, to teachers, as well as coinformants nominated by participants themselves. The joint South London and Maudsley and Institute of Psychiatry Research Ethics Committee approved each phase of the study. Parents gave informed consent, and twins gave assent between 5 and 12 years and then informed consent at age 18.

#### Dunedin cohort

Participants in the second cohort were members of the Dunedin Multidisciplinary Health and Development Study, a longitudinal investigation of health and behavior in a birth cohort (Fig. 1). Dunedin participants (*N* = 1,037; 91% of eligible births; 52% male) were all individuals born between April 1972 and March 1973 in Dunedin, New Zealand, who were eligible based on residence in the province and who participated in the first assessment at age 3. Full details about the sample are reported elsewhere (Poulton, Moffitt, & Silva, 2015). The cohort represented the full range of socioeconomic status (SES) in the general population of New Zealand’s South Island. On adult health, the cohort matches the New Zealand National Health and Nutrition Surveys on key health indicators (e.g., body mass index, smoking, visits to the doctor).

Assessments were carried out at birth and ages 3, 5, 7, 9, 11, 13, 15, 18, 21, 26, 32, and, most recently, 38 years, when 95% of the 1,007 participants still alive took part. At each assessment wave, participants are brought to the Dunedin research unit for a full day of interviews and examinations. These data are supplemented by searches of official records and by questionnaires that are mailed, as developmentally appropriate, to parents, teachers, and peers nominated by the participants themselves. The Otago Ethics Committee approved each phase of the study, and informed consent was obtained from all participants.

#### Genotyping and imputation

We used Illumina HumanOmni Express 12 BeadChip arrays (Version 1.1; Illumina, Hayward, CA) to assay common single-nucleotide polymorphism (SNP) variation in the genomes of cohort members. We imputed additional SNPs using the IMPUTE2 software (Version 2.3.1; https://mathgen.stats.ox.ac.uk/impute/impute_v2.html; Howie, Donnelly, & Marchini, 2009) and the 1000 Genomes Phase 3 reference panel (Abecasis et al., 2012). Imputation was conducted on autosomal SNPs appearing in dbSNP (Version 140; http://www.ncbi.nlm.nih.gov/SNP/; Sherry et al., 2001) that were “called” in more than 98% of the samples. Invariant SNPs were excluded. The E-Risk cohort contains MZ twins, who are genetically identical; we therefore empirically measured genotypes of one randomly selected twin per pair and assigned these data to their MZ cotwin. Prephasing and imputation were conducted using a 50-million-base-pair sliding window. The resulting genotype databases included genotyped SNPs and SNPs imputed with 90% probability of a specific genotype among the European-descent members of the E-Risk cohort (*N* = 1,999 participants in 1,011 families) and the non-Maori members of the Dunedin cohort (*N* = 918). We analyzed SNPs in Hardy-Weinberg equilibrium (*p* > .01).

### Polygenic scoring

Polygenic scoring was conducted following the method described by Dudbridge (2013) using PRSice (Euesden, Lewis, & O’Reilly, 2015). Briefly, SNPs reported in the results of the most recent genome-wide association study released by the Social Science Genetic Association Consortium (Okbay et al., 2016) were matched with SNPs in the E-Risk and Dunedin databases. For each SNP, the count of education-associated alleles was weighted according to the effect estimated in the genome-wide association study. Weighted counts were averaged across SNPs to compute polygenic scores. We used all matched SNPs to compute polygenic scores irrespective of nominal significance for their association with educational attainment and linkage disequilibrium between SNPs. (see Table S1 in the Supplemental Material available online for results from analyses of polygenic scores computed using a clumping approach that takes linkage disequilibrium into account. The pattern of findings was similar to using nonclumped scores.)

To control for possible population stratification, we conducted a principal component analysis of our genome-wide SNP database using PLINK (Version 1.9; Chang et al., 2015). Analyses were conducted separately in the E-Risk and Dunedin databases. In the E-Risk database, one twin was selected at random from each family for principal component analysis. SNP loadings for principal components were applied to cotwin genetic data to compute principal component values for the full sample. The 10 principal components explained 2.8% of variance in the education polygenic score in the E-Risk cohort and 1.2% in the Dunedin cohort. Within each database, we residualized polygenic scores for the first 10 principal components estimated from the genome-wide SNP data. The residualized scores were normally distributed. We standardized residuals (*M* = 0, *SD* = 1) for analysis. In replication of the genome-wide association study from which our education polygenic score was derived (Okbay et al., 2016), the score predicted educational attainment, measured as the highest degree completed at the time of the age-18 assessment in the E-Risk cohort (β = 0.21, 95% confidence interval, or CI = [0.16, 0.26], *R*^2^ = .045) and the age-38 assessment in the Dunedin cohort (β = 0.17, 95% CI = [0.10, 0.23], *R*^2^ = .028). For analyses where we reported the effects of having a “lower” polygenic score, we reverse-coded the score, so that higher numbers indicate a lower polygenic score for education (i.e., a greater genetic risk for low educational attainment).

### Criminal offending and trajectories of antisocial behavior

In the E-Risk cohort, official records of participants’ criminal offending were obtained through UK Police National Computer (PNC) record searches conducted in cooperation with the UK Ministry of Justice. Records include complete histories of cautions and convictions for participants in the United Kingdom beginning at age 10 years, the age of criminal responsibility. Our data are complete through age 19 years. Criminal offending was recoded into a binary variable to reflect whether participants had been cautioned or convicted or not.

In the Dunedin cohort, information on officially recorded criminal offending was obtained by searching the central computer system of the New Zealand Police, which provides details of all New Zealand convictions and sentences and Australian convictions communicated to the New Zealand Police. Searches were completed following each assessment, at ages 18, 21, 26, 32, and 38 (last search completed in 2013). Official records of criminal conviction were available from 14 years of age onward, the age from which criminal conviction for all types of offenses was permissible. Criminal offending was recoded into a binary variable to reflect whether participants had been convicted or not.

Using data from the Dunedin cohort, we supplemented the analyses by studying developmental trajectories of parent, teacher, and self-reported antisocial behavior from childhood to adulthood. These trajectories in the Dunedin cohort have been developed and described in previous articles about antisocial behavior in the Dunedin cohort (Odgers et al., 2007; Odgers et al., 2008). Briefly, antisocial conduct problems were assessed at ages 7, 9, 11, 13, 15, 18, 21, and 26 years through scoring six key symptoms of *Diagnostic and Statistical Manual of Mental Disorders* (4th ed.) conduct disorder as being present or absent at each age according to reports from parents, teachers, and study members: physical fighting, bullying others, destroying property, telling lies, truancy, and stealing (American Psychiatric Association, 1994). Symptoms were adapted across the age span to ensure that the measures were developmentally appropriate (e.g., work absenteeism was substituted for truancy at older ages). Growth mixture modeling was used to identify subgroups of participants that followed unique trajectories in their antisocial behavior over time. A four-class model represented the best empirical fit to the data according to several indices of model fit and classification accuracy. Across gender, the majority of participants (50%) displayed low levels of antisocial behavior across time (the “always-low” group), 22% exhibited antisocial behavior only in childhood (“childhood-limited”), 19% were characterized by adolescent-limited antisocial behavior (“adolescent-limited”), and 9% displayed persistently high levels of antisocial behavior across the years (“life-course persistent”). A recent follow-up study in the Dunedin cohort validated this classification, showing that life-course persistent cohort members averaged more convictions between ages 26 and 38 years compared with adolescent-limited cohort members, of whom very few received any convictions after age 26 (Rivenbark et al., 2017).

### Potential explanatory variables

We evaluated three sets of explanatory variables in both cohorts. Psychometric details about these measures are provided in previous publications.

#### Criminogenic family environment

In the E-Risk cohort, *SES* was defined using a standardized composite of parents’ income, education, and social class (Trzesniewski, Moffitt, Caspi, Taylor, & Maughan, 2006). We reverse-coded the variable to reflect socioeconomic deprivation. *Parental antisocial behavior* was assessed as father’s and mother’s antisocial personality (Caspi et al., 2001), reported by mothers using the Young Adult Behavior Checklist (Achenbach, 1997), modified to obtain lifetime data and supplemented with questions from the Diagnostic Interview Schedule (Robins, Cottler, Bucholz, & Compton, 1995). Reports about parents’ antisocial behavior were averaged and standardized (*M* = 0, *SD* = 1).

In the Dunedin cohort, SES was measured using a 6-point scale that assessed parents’ occupational statuses, defined using average income and educational levels derived from the New Zealand Census. Parents’ occupational statuses were assessed when participants were born and again at subsequent assessments up to age 15 years. The highest occupational status of either parent was averaged across the childhood assessments (Poulton et al., 2002), and the variable was standardized (*M* = 0, *SD* = 1) and reverse-coded to reflect socioeconomic deprivation. Parental antisocial behavior was assessed as father’s and mother’s history of antisocial behavior, using items from the Diagnostic Interview Schedule (Robins et al., 1995), reported by mothers and fathers (Milne et al., 2008). Reports about parents’ antisocial behavior were averaged and standardized (*M* = 0, *SD* = 1).

#### Poor educational qualifications

In the E-Risk cohort, *poor educational qualifications* were assessed by whether participants did not obtain or scored a low average grade (Grade D–G) on their General Certificate of Secondary Education (GCSE; 21.9% of participants). GSCEs are a standardized examination taken at the end of compulsory education at age 16 years.

In the Dunedin cohort, poor educational qualifications were assessed by whether participants did not sit their English or Math school certificate exam, a national examination held at about 15 years of age that was, at the time the Dunedin cohort was growing up, the most basic educational qualification in New Zealand (17.3% of participants).

#### Early-emerging psychological and behavioral risk factors

In the E-Risk cohort, participants’ *cognitive ability* was individually assessed at age 5 using a short form of the Wechsler Preschool and Primary Scale of Intelligence–Revised (WPPSI-R; Wechsler, 1990) comprising Vocabulary and Block Design subtests. IQ scores were prorated (i.e., the full-scale IQ score was estimated from two subscales) following procedures described by Sattler (1992, pp. 998–1004). IQ scores were standardized (*M* = 0, *SD* = 1). We reverse-coded the variable to reflect low cognitive ability. Participants’ *low self-control* during their first decade of life was measured using a multioccasion/multi-informant strategy, following Moffitt et al. (2011). Briefly, a self-control factor was estimated via multiple measures, including observational ratings of participants’ lack of control (age 5 years), parent and teacher reports of poor impulse control (ages 5, 7, and 10 years), self-reports of inattentive and impulsive behavior (age 7 years), and interviewer judgments of the personality trait of conscientiousness (age 10 years). On the basis of principal component analysis, we averaged the standardized measures into a single composite score (*M* = 0, *SD* = 1). Participants’ *academic performance* was assessed using teacher reports when participants were 7 and 10 years old. Teachers were asked to rate participant’s performance in English and Math on a 5-point scale ranging from 1, *far below average*, to 5, *far above average*. Scores were averaged across academic subjects and age and standardized (*M* = 0, *SD* = 1). We reverse-coded the variable so higher scores reflected more academic difficulties. *Truancy* was assessed using mother and teacher reports at ages 7 and 10. Truancy was considered present if either mothers or teachers reported truancy at either age (3.2%).

In the Dunedin cohort, participants’ cognitive ability was individually assessed at ages 7, 9, and 11 years using the Wechsler Intelligence Scale for Children–Revised (WISC-R; Wechsler, 1974). Scores were averaged across age and standardized (*M* = 0, *SD* = 1). We reverse-coded the variable to reflect low cognitive ability. Participants’ low self-control was measured using multiple measures of self-control: observational ratings of participants’ lack of control (ages 3 and 5) and parent, teacher, and self-reports of impulsive aggression, overactivity, lack of persistence, inattention, and impulsivity (ages 5, 7, 9, and 11; Moffitt et al., 2011). On the basis of principal component analysis, we averaged the standardized measures into a single composite score (*M* = 0, *SD* = 1). Participants’ academic performance was assessed at ages 7 and 9, when mothers were asked to rate their children’s performance in reading, printing, arithmetic, and spelling on a 3-point scale ranging from 0, *slow*, to 2, *above average*. Mothers’ responses were averaged across age and standardized (*M* = 0, *SD* = 1). We reverse-coded the variable to reflect academic difficulties. Truancy was assessed using mother and teacher reports at ages 7, 9, and 11. Truancy was considered present if mothers or teachers reported truancy at any age (7.7%).

### Statistical analyses

We used liability threshold models to estimate genetic, shared environmental, and nonshared environmental influences on criminal offending in the E-Risk cohort. We used Poisson regression models with robust standard errors to estimate relative risks for the binary dependent outcome of having a criminal record and to investigate whether criminogenic family environment, poor educational qualifications, and early-emerging risk factors could explain the effects. Formal mediation analysis for binary outcomes, as implemented in Stata, was used to test whether poor educational qualifications and early-emerging risk factors accounted for a significant portion of the genetic association with offending. In the mediation analyses, 95% confidence intervals were obtained from 500 bootstrap replications; in the E-Risk cohort, this was done accounting for the clustering of the twin data. We used survival analysis to test whether participants with lower versus higher polygenic scores tended to become convicted earlier in life and multinomial logistic regression to estimate relative risks for membership in different developmental trajectories of antisocial behavior. Both twins were included in the analyses of the E-Risk cohort; we accounted for nonindependence of observations of twins within families by clustering standard errors at the family level. We also repeated the main analyses including only one randomly selected twin of each pair in the E-Risk cohort (see Table S2 in the Supplemental Material). *R*^2^ and pseudo-*R*^2^-based measures of effect size for all outcomes are reported in Table S3 in the Supplemental Material.

## Results

### Are there genetic influences on official records of offending?

Official records of participants’ cautions and convictions were obtained through national police record searches through age 19 years in the E-Risk cohort and age 38 years in the Dunedin cohort. Using the E-Risk twin design, we first sought to replicate findings from previous twin and adoption studies of quantitative genetic influences on criminal offending to establish that there was a basis to proceed with our analyses of testing for an association between a molecular-based polygenic score and offending. MZ twins in the E-Risk cohort were more similar in their criminal offending (tetrachoric correlation *r* = .81, 95% CI = [.71, .90]) than were DZ twins (*r* = .61, 95% CI = [.44, .77]), indicating genetic influences. A univariate liability threshold model was used to estimate genetic, shared environmental, and nonshared environmental influences on criminal offending. Genetic influences accounted for an estimated 41% (95% CI = [5, 81]) of the variance in offending in the E-Risk cohort; 40% of the variance was accounted for by shared environmental influences (95% CI = [2, 70]) and 19% by nonshared environmental influences (95% CI = [11, 30]). These estimates are consistent with meta-analytic findings from twin studies of antisocial behavior (Rhee & Waldman, 2002).

### Do participants’ polygenic scores predict their official records of offending?

**Fig. 2.**
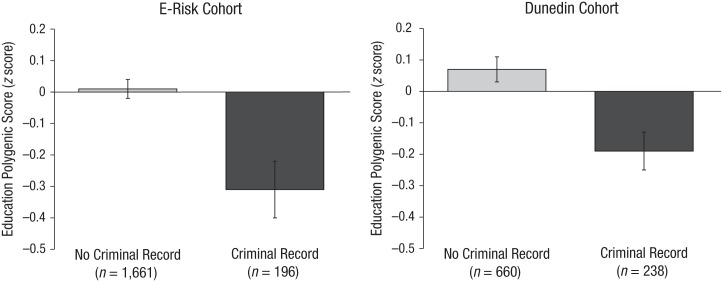
Mean education polygenic score among participants with and without a criminal record, through age 19 years in the Environmental Risk (E-Risk) cohort and age 38 years in the Dunedin cohort. Error bars reflect standard errors, with robust standard errors in the E-Risk cohort. E-Risk and Dunedin participants with a criminal record had lower polygenic scores for education than participants without a criminal record.

We next tested the hypothesis that molecular-genetic predictors of educational attainment would forecast participants’ criminal offending. In both cohorts, participants with lower polygenic scores for educational attainment were at greater risk to grow up to have a criminal record. The increase in risk was modest: a standard-deviation decrease in the polygenic score was associated with a 20% to 30% greater risk of having been cautioned or convicted (E-Risk cohort: incidence-rate ratio, or IRR = 1.33, 95% CI = [1.13, 1.55], *p* < .01; Dunedin cohort: IRR = 1.21, 95% CI = [1.09, 1.34], *p* < .01; Table 1). Effect sizes were similar across the two cohorts (Fig. 2) and across sex (males: IRR = 1.25, 95% CI = [1.13, 1.39], *p* < .01; females: IRR = 1.30, 95% CI = [1.08, 1.57], *p* < .01, with participants pooled across cohorts). Results also showed that although having a low polygenic score for educational attainment increased the risk of officially recorded offending, even among individuals with very low scores, the majority had no criminal record (see Table S4 in the Supplemental Material).

**Table 1.** Incidence-Rate Ratios for the Association Between Polygenic Scores for Educational Attainment and Criminal Offending in the Two Birth Cohorts

Effect and variable	E-Risk cohort (*n* = 1,857)^a^		Dunedin cohort (*n* = 898)^a^	
	Bivariate	Multivariate	Bivariate	Multivariate
Effect of participants’ polygenic scores on their official records of offending				
Low polygenic score for educational attainment^b^	1.33 [1.13, 1.55]	—	1.21 [1.09, 1.34]	—
Polygenic effect on offending, accounting for a criminogenic family environment				
Low polygenic score for educational attainment^b^	1.34 [1.14, 1.57]	1.20 [1.01, 1.42]	1.23 [1.11, 1.36]	1.17 [1.05, 1.30]
Socioeconomic deprivation	2.26 [1.88, 2.71]	1.96 [1.58, 2.44]	1.40 [1.25, 1.57]	1.35 [1.19, 1.52]
Parental antisocial behavior	1.49 [1.34, 1.66]	1.18 [1.03, 1.35]	1.16 [1.05, 1.27]	1.07 [0.96, 1.18]
Polygenic effect on offending, accounting for leaving school with poor qualifications				
Low polygenic score for educational attainment^b^	1.33 [1.13, 1.56]	1.19 [1.02, 1.39]	1.21 [1.09, 1.34]	1.14 [1.03, 1.26]
Leaving school with poor qualifications	4.27 [3.27, 5.59]	4.02 [3.05, 5.28]	2.83 [2.31, 3.46]	2.71 [2.21, 3.32]
Polygenic effect on offending, accounting for early-emerging psychological and behavioral risk factors				
Low polygenic score for educational attainment^b^	1.30 [1.11, 1.53]	1.22 [1.05, 1.42]	1.21 [1.09, 1.35]	1.14 [1.02, 1.27]
Low cognitive ability	1.58 [1.35, 1.85]	1.11 [0.93, 1.32]	1.27 [1.14, 1.43]	.99 [0.86, 1.13]
Low self-control	2.06 [1.83, 2.32]	1.80 [1.58, 2.04]	1.54 [1.43, 1.67]	1.48 [1.34, 1.64]
Academic difficulties in primary school	1.68 [1.47, 1.93]	1.17 [1.00, 1.37]	1.30 [1.16, 1.45]	1.02 [0.89, 1.17]
Truancy	3.92 [2.65, 5.80]	1.98 [1.38, 2.83]	2.11 [1.61, 2.76]	1.25 [0.91, 1.72]

Note: Values in brackets are 95% confidence intervals.

a Crime records data were obtained for 93% (1,857/1,999) and 98% (898/918) of the Environmental Risk (E-Risk) and Dunedin cohorts, respectively. ^b^The polygenic score was reverse-coded in these analyses, so that a higher score indicates a greater genetic risk for low educational attainment.

### Is the influence of participants’ polygenic scores on future offending accounted for by criminogenic family environment?

We next examined whether genetic influences increased risk for officially recorded offending independently of a criminogenic family environment, as indicated by growing up in socioeconomic deprivation and having parents who display antisocial behavior. We conducted this test for two reasons. First, we aimed to rule out the possibility that genetic associations with criminal offending solely reflected gene-environment correlations, whereby parents pass on genetic variants for low educational attainment to their children and also create an environment that increases their children’s risk of offending. Second, we aimed to test whether polygenic scores predicted offending over and above two well-established, global predictors of criminal offending that contain both genetic and environmental influences. In both cohorts, Poisson regression models indicated that, as expected, participants who grew up in socioeconomically deprived families and who had parents who displayed antisocial behavior were at greater risk to have a criminal record (Table 1, bivariate models). We also observed a correlation between participants’ polygenic scores and these two features of the environments they grew up in. Participants with lower polygenic scores for education were more likely to have grown up in socioeconomically deprived households (E-Risk cohort: *r* = .23, 95% CI = [.17, .29], *p* < .01; Dunedin cohort: *r* = .16, 95% CI = [.09, .22], *p* < .01) and with parents who displayed antisocial behavior (E-Risk cohort: *r* = .06, 95% CI = [.01, .11], *p* < .05; Dunedin cohort: *r* = .13, 95% CI = [.06, .19], *p* < .01). After accounting for these two indicators of criminogenic family environment by including them in the model as covariates, participants’ polygenic scores continued to forecast their criminal offending (Table 1, multivariate models).

### Is the effect of polygenic scores accounted for by leaving school with poor qualifications?

**Fig. 3.**
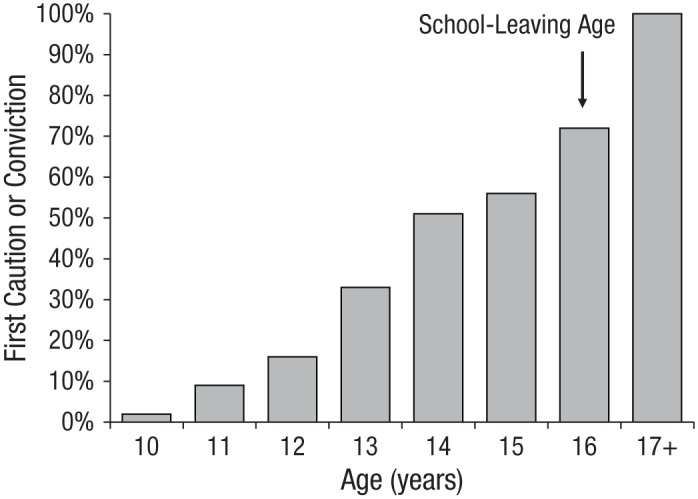
Cumulative distribution of the first appearance in police records of cautions and convictions, by age, of the 196 participants with criminal records in the Environmental Risk (E-Risk) cohort. In the United Kingdom, compulsory schooling ends at age 16 years. The majority of E-Risk participants with criminal records received their first caution or conviction before school-leaving age.

We investigated characteristics that may connect genetic differences between individuals with official records of criminal offending. Because the polygenic score we used comes from a genome-wide association study of educational attainment, we first tested whether it predicted offending because it was associated with poor educational qualifications. Our findings provided some support for poor educational qualifications as an explanation for the link between polygenic scores for educational attainment and offending. Participants with lower polygenic scores were more likely to leave school with poor educational qualifications in both the E-Risk cohort (polychoric *r* = .21, 95% CI = [.13, .28], *p* < .01) and the Dunedin cohort (polychoric *r* = .19, 95% CI = [.09, .29], *p* < .01). Poor educational qualifications, in turn, were associated with criminal offending (Table 1, bivariate models). Once poor qualifications were included as a covariate in the model, the effect of polygenic scores on offending was reduced (Table 1, multivariate models). Formal mediation analyses indicated that poor qualifications accounted for a significant portion of the genetic association with offending (see Table S5 in the Supplemental Material). However, two results indicate that there is more to the observed genetic effect than poor educational qualifications alone. First, the polygenic score continued to predict offending in both cohorts after qualifications were included in the model (Table 1, multivariate models). Second, in the E-Risk cohort, cautions and convictions are recorded from age 10 onward; examining their cumulative distribution across ages revealed that the majority of participants with criminal records received their first caution or conviction before the end of compulsory schooling (Fig. 3). This finding indicates that polygenic influences predicting offending already manifested earlier in life, while participants were still in school. We therefore turned our attention to the first decade of life.

### Are influences of participants’ polygenic scores on future offending accounted for by early-emerging psychological and behavioral risk factors?

Our findings indicated that part of the reason why participants with lower polygenic scores are at greater risk to become involved in crime is that they display a constellation of psychological and behavioral risk factors for school problems as well as offending from a young age. As children, participants with lower polygenic scores for educational attainment exhibited lower cognitive ability (E-Risk cohort: *r* = .14, 95% CI = [.09, .19], *p* < .01; Dunedin cohort: *r* = .22, 95% CI = [.15, .29], *p* < .01), poorer self-control (E-Risk cohort: *r* = .06, 95% CI = [.00, .11], *p* = .050; Dunedin cohort: *r* = .13, 95% CI = [.06, .20], *p* < .01), more academic difficulties in primary school (E-Risk cohort: *r* = .14, 95% CI = [.08, .20], *p* < .01; Dunedin cohort: *r* = .19, 95% CI = [.12, .25], *p* < .01), and in the Dunedin cohort, more truancy (E-Risk cohort: *r* = .08, 95% CI = [−.05, .20], *p* = .19; Dunedin cohort: *r* = .15, 95% CI = [.03, .28], *p* < .01). Each of these risk factors in turn predicted offending (Table 1, bivariate models). Including all early-emerging risk factors in the same model reduced the prediction of participants’ polygenic scores on criminal records in both cohorts (Table 1, multivariate models). Formal mediation analyses indicated that early-emerging risk factors accounted for a significant portion of the genetic association with offending (see Table S5 in the Supplemental Material).

### Do polygenic scores predict the timing and persistence of antisocial behavior across the life course?

**Fig. 4.**
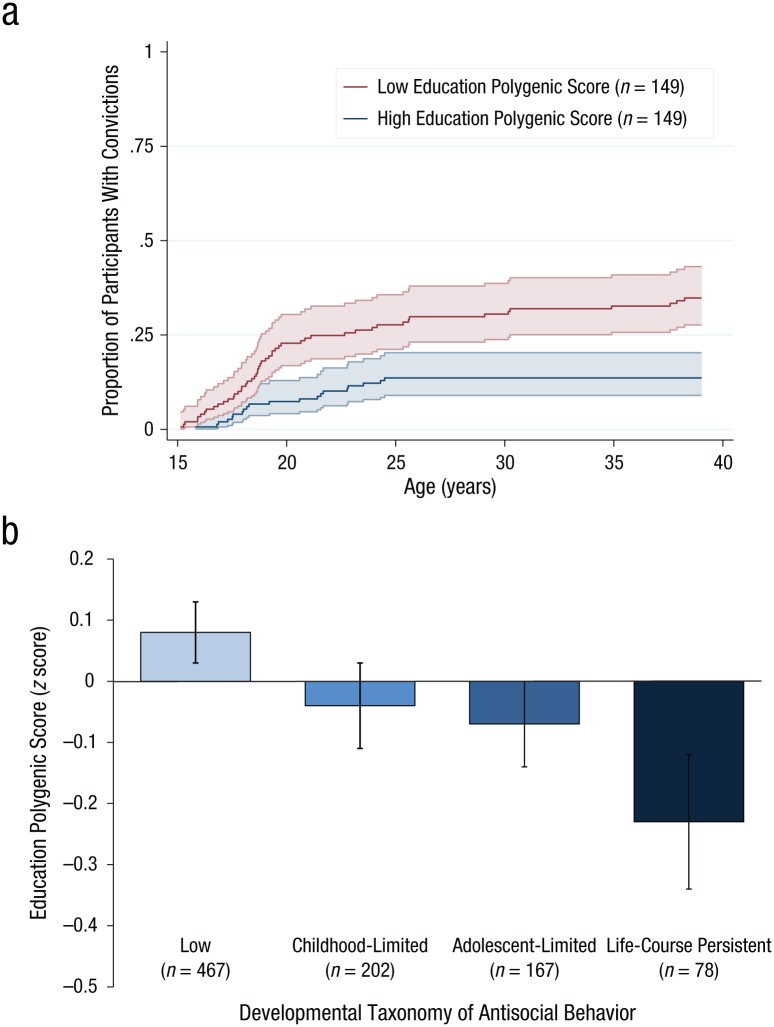
Association between polygenic score for education and the timing and persistence of antisocial behavior across the life course. Panel (a) depicts the proportion of Dunedin participants with convictions by age (Kaplan Meier failure functions) among participants with low (< 1 *SD* below the mean) and high (> 1 *SD* above the mean) polygenic scores for educational attainment. Shaded areas show 95% confidence intervals. Panel (b) shows mean differences in polygenic score across four developmental subtypes of antisocial behavior in the Dunedin cohort, following Moffitt (1993b). Error bars reflect standard errors.

In the Dunedin cohort, we first analyzed whether lower polygenic scores for educational attainment predicted an earlier onset of offending. Survival analyses indicated that participants with lower education polygenic scores tended to get convicted earlier in life (hazard ratio = 1.25, 95% CI = [1.10, 1.42], *p* < .01; Fig. 4a). Second, we tested whether polygenic risk was particular to the life-course-persistent pattern of antisocial behavior, as opposed to shorter-term involvement. We analyzed life-course trajectories of antisocial behavior as reported by parents, teachers, and participants themselves from childhood to adulthood by comparing the polygenic scores of participants classified in our previous research into four developmental subtype groups according to their longitudinal pattern of participation in antisocial behavior: always-low, childhood-limited, adolescent-limited, and life-course persistent (Fig. 4b). Results from multinomial regression models supported our hypothesis that participants with lower polygenic scores would be significantly more likely to belong to the life-course-persistent subtype than to the always-low-antisocial subtype (relative-risk ratio, or RRR = 1.36, 95% CI = [1.07, 1.73], *p* < .05). As also hypothesized, participants with lower polygenic scores were not at significantly greater risk of belonging to the childhood-limited or adolescent-limited subtype, relative to the always-low-antisocial subtype (RRR = 1.13, 95% CI = [0.95, 1.33], *p* = .16, and RRR = 1.16, 95% CI = [0.97, 1.39], *p* = .10, respectively).

## Discussion

In two birth cohorts of individuals growing up 20 years and 20,000 kilometers apart, we tested the hypothesis that molecular-genetic predictors of educational attainment, summarized in a polygenic score, would predict criminal offending. We chose to examine a polygenic score derived from genome-wide association studies of education because low educational attainment and criminal offending are linked through established criminological theories. Our findings revealed that participants with lower polygenic scores for educational attainment were more likely to have a criminal record by midlife. This effect was small in size but robust across replication. Although we observed a gene-environment correlation, whereby individuals with lower polygenic scores were more likely to grow up in criminogenic environments, genetic associations remained after accounting for familial predictors of offending, including socioeconomic deprivation and parental antisocial behavior. Early-emerging psychological and behavioral risk factors for school problems and crime, including low cognitive ability, poor self-control, academic difficulties and truancy, connected differences in DNA with participants’ later criminal offending. The effect of participants’ polygenic scores extended to the onset of their criminal offending and trajectories of antisocial behavior across the life course, as indexed by developmental trajectories of life-course-persistent antisocial behavior. Taken together, the findings show how polygenic discoveries for educational attainment can be used to study pathways leading from genes to offending. They also suggest that early-emerging risk factors that influence whether children have a good or bad experience of school may serve as intervention targets to prevent some of the genetic influences on offending from unfolding.

Three aspects of the present study further bolster the substance of our finding of a link between a polygenic score for educational attainment and offending. First, our analyses across two population-representative cohorts revealed that the findings were robust, answering calls for reproducibility in psychological and genomic science. Second, retention rates in both cohorts are high (93% and 95% in the E-Risk and Dunedin cohorts, respectively), reducing the risk of biased estimates when examining behaviors, such as offending, that are susceptible to attrition. Third, in both cohorts, the research linked participants’ genetic information with electronic crime records. Although crime records underreport offending, they have the advantages of being less susceptible than self-reports to reporting biases, recall failure, and concealment. The approach of integrating genetic information with administrative records is increasingly being used to advance medical research (McCarty et al., 2011) but is not yet widely adopted in the social sciences.

It may seem surprising that genetic variants identified in a genome-wide association study for educational attainment predict criminal offending. However, this hypothesis was derived by incorporating theoretical accounts of crime causation with recent genomic discoveries about educational attainment. More generally, the findings illustrate how established social-science theories can guide the characterization of genomic discoveries for human behavior (Belsky, Moffitt, & Caspi, 2013).

Although we identified characteristics that mediated some of the influence of the polygenic score on offending, we could not explain all of the effect. Our inability to do so can fuel follow-up work. Polygenic scores may predict offending via early-emerging deficits in neurocognitive functioning, such as the ability to learn from rewards and punishments. Another possibility is that individuals with lower polygenic scores have deficits in systems that influence socioemotional processing, putting them at greater risk of experiencing difficulties in school as well as engaging in delinquent behavior.

The findings should be interpreted in light of limitations. First, polygenic influences shared with education may reflect only a small proportion of all genetic influences on crime and may exert their effects via different pathways. A recent genome-wide association study on antisocial behavior (Tielbeek et al., 2017) reported a genetic correlation (*r*) with educational attainment of −0.52, indicating both genetic overlap with educational attainment and unique genetic effects on crime. Whether or not the developmental processes and mechanisms driving shared and unique genetic effects are the same remains to be tested. Second, the findings cannot be generalized to individuals of non-European ancestry because allele frequencies, linkage disequilibrium patterns, and environmental moderators of the association may vary across populations (Rosenberg et al., 2010). Third, we analyzed official crime records, which reflect only a portion of offenders and offenses. Reassuringly, we obtained similar findings when analyzing trajectories of persistent conduct problems derived from parent, teacher, and self-reports. Fourth, the polygenic score for education accounted for only a small portion of variation in criminal offending, both in relative and absolute terms. The small effect size relative to other predictors (e.g., parental antisocial behavior, cognitive ability) is to be expected because these comprise both environmental and genetic influences. Against this background, it is remarkable that the polygenic score accounted for additional variance beyond traditional risk factors. As genome-wide association studies become larger, the proportion of variance accounted for by molecular-genetic variables will likely increase. Finally, there are additional hypotheses that can be tested using the polygenic score, which we have not examined. For example, we did not have enough statistical power to examine interactions between the polygenic score and SES, and we restricted our analyses to two broad indicators of antisocial behavior—criminal offending and life-course-persistent antisocial behavior—rather than zooming in on finer distinctions. We also did not test hypotheses about the interplay between children’s genes and aspects of their environment, such as parenting. Our priority was to conduct an initial examination of genetic links with “workhorse” phenotypes in criminology and to test robustness across cohorts. This research can now be taken forward to test further hypotheses in other, larger samples.

The findings have implications for public and scientific debates about genetic research on social and antisocial behavior. First, a key result from this and previous studies is that discoveries in genome-wide association studies of educational attainment are related not only to education but also to life-course success and adversity more generally (Belsky et al., 2016). These findings are in line with the notion of educational attainment as a proxy phenotype for related phenotypes (Rietveld et al., 2014). They also underscore the pervasiveness of pleiotropy (i.e., the phenomenon that genomic discoveries for one particular phenotype also predict related outcomes). Together with polygenicity (i.e., the observation that one outcome is influenced by many genes), findings of pleiotropy are moving sociogenomic research further away from a deterministic paradigm of “one gene one outcome” and toward an understanding that many genes affect many outcomes through their influences on early-emerging characteristics that shape life-course development.

Second, our findings hark back to the nature-nurture debate and the question of whether criminals are born or made (Wilson & Herrnstein, 1985). Using a polygenic scoring approach that overcomes lingering reservations about the validity of twin and adoption studies (Burt & Simons, 2014), we demonstrated that some children are born with genetic propensities that are associated with their risk to offend. However, our findings do not support a view of genetics as destiny. Many children who carry few education-associated alleles develop good behavioral control, complete schooling, and do not engage in delinquent behavior. Others develop behavioral problems, drop out of school, and become involved in crime. Alongside environmental factors, genetics explain a small proportion of these individual differences in life outcomes. Genetic risk operates through a series of intermediate phenotypes that are also under the influence of the social environment and that can provide targets for intervention, such as low self-control and academic difficulties. Intervening with these early-emerging characteristics and behaviors (e.g., through early training of self-control and academic skills; Heckman, 2006) may be one strategy to disrupt pathways from genes to offending.

Finally, some people look back at the fraught history of behavioral genetics and wonder whether genetic influences on social behavior should be studied at all. Instead of fearing sociogenomic research or focusing on genetics to the neglect of other risk factors, here we incorporated molecular genetic predictors into existing sociological and psychological theories and found that a polygenic score for education acts much like any other risk factor for offending: It has modest, probabilistic effects that are mediated by characteristics and behaviors criminologists have studied for decades. Our study demonstrates that existing theories in the social and behavioral sciences can accommodate molecular-genetic discoveries by weaving them into the frameworks of understanding that we already have about human behavior.

## Supplemental Material

WertzSupplementalMaterial – Supplemental material for Genetics and Crime: Integrating New Genomic Discoveries Into Psychological Research About Antisocial Behavior Click here for additional data file. Supplemental material, WertzSupplementalMaterial for Genetics and Crime: Integrating New Genomic Discoveries Into Psychological Research About Antisocial Behavior by J. Wertz, A. Caspi, D. W. Belsky, A. L. Beckley, L. Arseneault, J. C. Barnes, D. L. Corcoran, S. Hogan, R. M. Houts, N. Morgan, C. L. Odgers, J. A. Prinz, K. Sugden, B. S. Williams, R. Poulton, and T. E. Moffitt in Psychological Science
