# Supplementary material for: Genetics and Crime: Integrating New Genomic Discoveries Into Psychological Research About Antisocial Behavior
Source: Psychol Sci. 2018 Mar 7;29(5):791–803. doi: 10.1177/0956797617744542 (PMC5945301; doi:10.1177/0956797617744542)
Supplement: WertzSupplementalMaterial – Supplemental material for Genetics and Crime: Integrating New Genomic Discoveries Into Psychological Research About Antisocial Behavior [file WertzSupplementalMaterial.pdf]

SUPPLEMENTARY MATERIALS

Supplementary Table S1. Repeat of main analyses, using non-clumped and clumped polygenic scores.

	E-Risk cohort		Dunedin cohort	
	Not-clumped	Clumped	Not-clumped	Clumped
	IRR (95% CI)	IRR (95% CI)	IRR (95% CI)	IRR (95% CI)
Panel A. Effect of participants' polygenic scores on their official records of offending	1.33 (1.13, 1.55)	1.40 (1.19, 1.65)	1.21 (1.09, 1.34)	1.22 (1.10, 1.35)
Panel B. Polygenic effect on offending, accounting for a criminogenic family environment	1.20 (1.01, 1.42)	1.20 (1.00, 1.43)	1.17 (1.05, 1.30)	1.19 (1.07, 1.34)
Panel C. Polygenic effect on offending, accounting for leaving school with poor qualifications	1.19 (1.02, 1.39)	1.25 (1.07, 1.46)	1.14 (1.03, 1.26)	1.14 (1.03, 1.26)
Panel D. Polygenic effect on offending, accounting for early-emerging psychological and behavioral risk factors	1.22 (1.05, 1.42)	1.27 (1.08, 1.49)	1.14 (1.02, 1.27)	1.15 (1.04, 1.29)

Note: Clumped scores were computed using a 500kb window and an R^2 threshold of 0.1, following Ripke et al. (2014). Clumped polygenic scores were residualized for the first ten principal components and residuals were standardized to Mean=0, Standard deviation=1. We reverse-coded the score, so that a higher score indicates a greater genetic risk for low educational attainment.

Supplementary Table S2. Repeat of main analyses in the E-Risk cohort, using only one randomly selected twin per pair.

	E-Risk cohort
	IRR (95% CI)
Panel A. Effect of participants' polygenic scores on their official records of offending	1.44<br>(1.16, 1.79)
Panel B. Polygenic effect on offending, accounting for a criminogenic family environment	1.31<br>(1.04, 1.66)
Panel C. Polygenic effect on offending, accounting for leaving school with poor qualifications	1.27<br>(1.03, 1.58)
Panel D. Polygenic effect on offending, accounting for early-emerging psychological and behavioral risk factors	1.35<br>(1.10, 1.66)

Supplementary Table S3. R^2 and pseudo-R^2-based measures of effect-size for all outcomes.

	E-Risk cohort		Dunedin cohort	
Outcome	R^2	pseudo R^2	R^2	pseudo R^2
Offending	-	0.012	-	0.008
Socioeconomic deprivation	0.052	-	0.024	-
Parental antisocial behavior	0.004	-	0.015	-
Leaving school with poor qualifications	-	0.015	-	0.015
Low cognitive ability	0.019	-	0.044	-
Low self-control	0.003	-	0.015	-
Academic difficulties in primary school	0.019	-	0.035	-
Truancy	-	0.003	-	0.012

Note: R^2s are reported for continuous outcomes; pseudo R^2s are reported for dichotomous outcomes. R^2 estimates may be downwardly biased because of the substantial measurement error contained in polygenic scores. For a discussion of methods to correct for this attenuation, see Tucker-Drob (2017).

Supplementary Table S4. Participants cautioned or convicted among those with low, average, and high polygenic scores for educational attainment. Although the risk for a criminal record was increased among participants with low polygenic scores for education, the majority had no criminal record.

	E-Risk cohort	Dunedin cohort
	% cautioned or convicted (N)*	% cautioned or convicted (N)*
Low polygenic score for educational attainment	15.7% (50)	34.2% (51)
Average polygenic score for educational attainment	10.2% (128)	27.8% (167)
High polygenic score for educational attainment	6.3% (18)	13.4% (20)

*The % and N express the percentage and number of people with a criminal record, among participants with low (<1 *SD* below the mean), average (within 1 *SD* of mean), and high (>1 *SD* above the mean) polygenic scores for educational attainment.

Supplementary Table S5. Formal mediation analyses in the E-Risk and Dunedin cohorts.

	E-Risk cohort	Dunedin cohort
	Estimate (95% CI)*	Estimate (95%CI)*
Mediator model (Poor qualifications)		
Total effect	.18 (.08, .28)	.16 (.07, .24)
Direct effect	.11 (.01, .21)	.10 (.02, .18)
Total indirect effect	.07 (.04, .10)	.06 (.03, .09)
% Mediation	39%	38%
Mediator model (Low cognitive ability, low self-control, academic difficulties, truancy)		
Total effect	.17 (.07, .26)	.16 (.07, .24)
Direct effect	.12 (.03, .21)	.11 (.02, .19)
Total indirect effect	.05 (.02, .08)	.05 (.01, .09)
% Mediation	29%	31%

* Indicates standardized estimates of total, direct and indirect effects in mediation models. 95% confidence intervals (CI) were obtained from 500 bootstrap replications; in E-Risk, this was done accounting for the clustering of the twin data.

References

Ripke, S., Neale, B. M., Corvin, A., Walters, J. T., Farh, K. H., Holmans, P. A., ... & Pers, T. H. (2014). Biological insights from 108 schizophrenia-associated genetic loci. *Nature*, *511*, 421-427.

Tucker-Drob, E. (2017). Measurement error correction of genome-wide polygenic scores in prediction samples. *BioRxiv*.
